# PTBP1 functions as a suppressor of ferroptosis in endometrial carcinoma cells by stabilizing SLC7A11 mRNA

**DOI:** 10.1007/s12672-025-04128-0

**Published:** 2025-11-28

**Authors:** Keke Zhu, Ke Zhang, Hui Li, Yao Shen, Lu Wang, Qinghong Hu, Hanwen Xing, Liping Han

**Affiliations:** 1 Department of Obstetrics and Gynecology, The First Affiliated Hospital of Zhengzhou University, No. 1 Jianshe East Road, Erqi District, Zhengzhou, 450000 Henan China; 2 Department of Obstetrics and Gynecology, Jiaozuo City People’s Hospital, NO. 267, Jiefang Road, Jiaozuo, 454150 Henan China; 3 Department of Oncology, Jiaozuo City People’s Hospital, NO. 267, Jiefang Road, Jiaozuo, 454150 Henan China; 4 Department of Supply Room, Jiaozuo City People’s Hospital, NO. 267, Jiefang Road, Jiaozuo, 454150 Henan China; 5 Department of Molecular Laboratory, Jiaozuo City People’s Hospital, NO. 267, Jiefang Road, Jiaozuo, 454150 Henan China; 6 Department of Radiotherapy, The First Affiliated Hospital of Zhengzhou University, No. 1 Jianshe East Road, Erqi District, Zhengzhou, 450000 Henan China

**Keywords:** Ferroptosis, Endometrial carcinoma, RNA binding protein, PTBP1, mRNA stability, SLC7A11

## Abstract

The RNA-binding protein polypyrimidine tract-binding protein 1 (PTBP1) facilitates tumor progression in various cancers by interacting with mRNAs of key oncogenes and tumor suppressors, thereby regulating their mRNA stability, alternative splicing, and protein translation. In this study, we found that​​ PTBP1 knockdown notably inhibited both in vitro cell viability and in vivo tumor growth in endometrial cancer (EC) cells. Elevated PTBP1 protein levels were also observed in endometrial cancer tissues compared to normal endometrial tissues. Transcriptomic sequencing revealed that PTBP1 depletion might impact lipid metabolism and ferroptosis in endometrial cancer cells. Through measurements of intracellular reactive oxygen species (ROS), glutathione (GSH), malondialdehyde (MDA), and Fe^2+^ levels, along with the expression of ferroptosis-related genes [acyl-CoA synthetase long-chain family member 4 (ACSL4), NADPH oxidase 1 (NOX1), glutathione peroxidase 4 (GPX4), and heat shock protein 27 (HSP27)], it was demonstrated that PTBP1 knockdown significantly enhances ferroptosis in endometrial cancer cells. Mechanistic studies further indicated that PTBP1 binds to the 5’ untranslated region (UTR) of solute carrier family 7 member 11 (SLC7A11) mRNA, stabilizing it and preserving SLC7A11 protein expression, thus inhibiting ferroptosis. Our study proposes a molecular mechanism by which PTBP1 promotes endometrial cancer progression by suppressing ferroptosis​​, suggesting that PTBP1 may be a promising therapeutic target for endometrial cancer.

**Supplementary Information:**

The online version contains supplementary material available at 10.1007/s12672-025-04128-0.

## Highlights

1. PTBP1 disruption induces ferroptosis in EC cells.
2. PTBP1 stabilizes SLC7A11 mRNA.
3. PTBP1 regulates EC cell ferroptosis *via *SLC7A11.

**Graphical Abstract:**

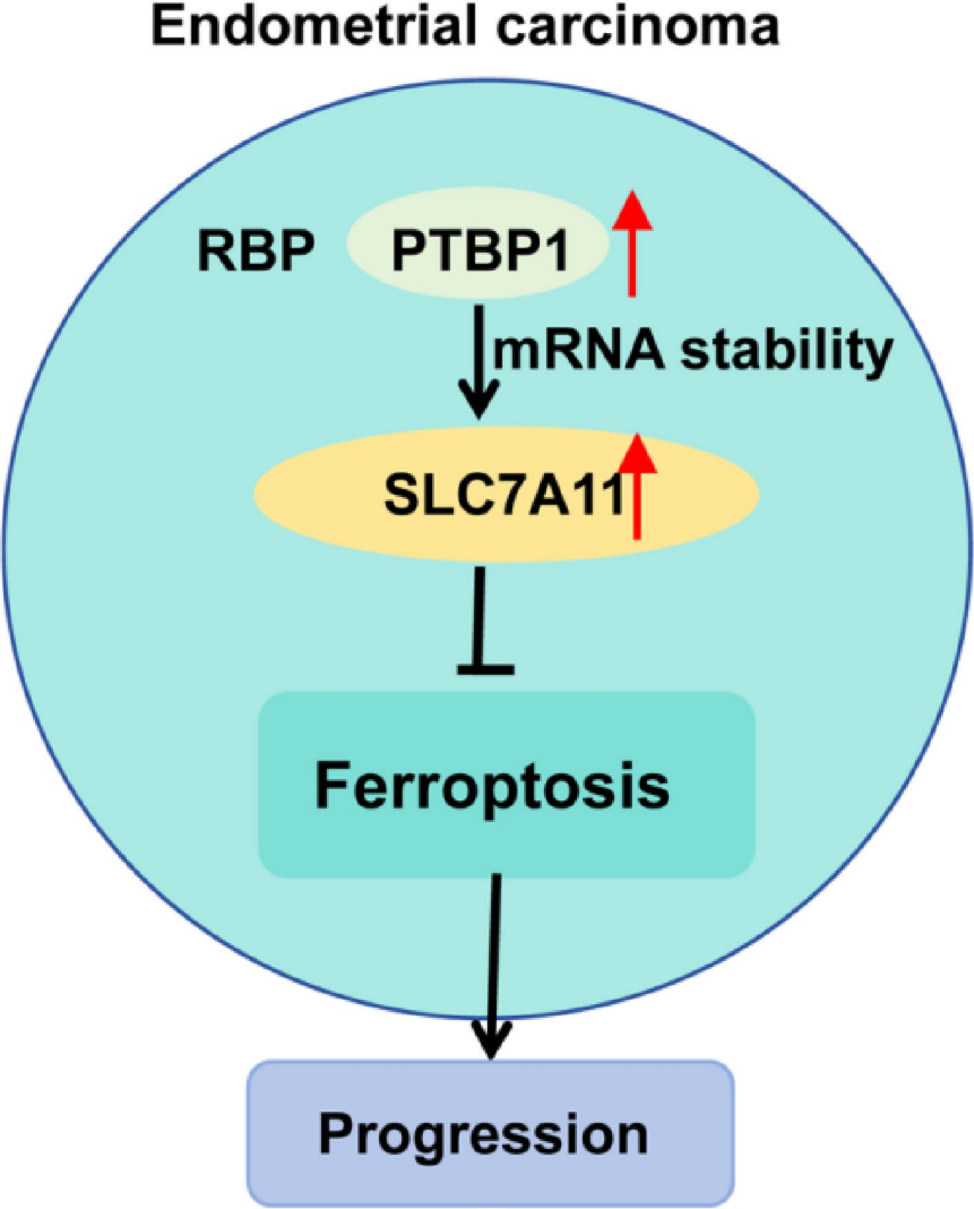

**Supplementary Information:**

The online version contains supplementary material available at 10.1007/s12672-025-04128-0.

## Introduction

Endometrial carcinoma (EC) is one of the most prevalent malignancies of the female reproductive system, with its incidence steadily rising in recent years [1–3]. While early-stage patients can achieve favorable outcomes through surgery and adjuvant therapies, treatment options for advanced or recurrent cases remain limited, resulting in significantly reduced survival rates [1, 4–6]. Consequently, a deeper understanding of the pathological mechanisms of endometrial carcinoma and the identification of novel therapeutic targets have become imperative.

Ferroptosis, an iron-dependent form of programmed cell death triggered by lipid peroxidation [7, 8], has emerged as a promising therapeutic avenue due to its involvement in critical cancer progression processes including tumorigenesis, development, and therapy resistance [7, 9, 10]. This distinct cell death modality is characterized by intracellular iron accumulation, glutathione (GSH) depletion, inactivation of glutathione peroxidase 4 (GPX4), and consequent accumulation of lipid peroxides [7, 8, 11]. The cystine/glutamate antiporter solute carrier family 7 member 11 (SLC7A11) serves as a core regulator of ferroptosis by mediating cystine uptake for GSH synthesis, thereby suppressing lipid peroxidation and ferroptosis execution [12, 13]. SLC7A11 is highly expressed in various cancers, including endometrial carcinoma, where its expression correlates with poor patient prognosis [14, 15]. While epigenetic mechanisms such as BRCA1-associated protein 1 (BAP1)-mediated H2Aub deubiquitination and lysine demethylase 4 A (KDM4A)-regulated H3K9me3 methylation can influence SLC7A11 transcription [16, 17], post-transcriptional regulation, particularly the control of SLC7A11 mRNA stability, represents a crucial yet underexplored regulatory layer for this key ferroptotic brake [18, 19]. Previous bioinformatics analyses have linked ferroptosis-related gene expression, including SLC7A11, to EC progression [20], yet experimental validation of the specific RNA-binding proteins (RBPs) governing SLC7A11 mRNA stability in EC remains lacking.

This knowledge gap directs attention to RBPs as master regulators of post-transcriptional gene expression. Polypyrimidine tract-binding protein 1 (PTBP1), a well-characterized RBP, promotes tumor progression in various cancers by modulating mRNA splicing, stability, and translation, with established roles in metabolic reprogramming and drug resistance [21–23]. ​​Notably, emerging evidence begins to implicate PTBP1 in ferroptosis regulation, though its functional impact appears strikingly context-dependent. In non-cancerous pathological models such as acute lung injury, PTBP1 regulates ferroptosis by enhancing the stability of acyl-CoA synthetase long-chain family member 4 (ACSL4) mRNA [24]. In liver cancer, PTBP1 modulates ferroptosis through a mechanism involving nuclear receptor coactivator 4 (NCOA4) [25]. These divergent findings highlight the cell-type-specific nature of PTBP1’s function in ferroptosis, raising the critical question of its role in EC.​​ Given PTBP1’s documented ability to stabilize mRNAs encoding critical metabolic proteins [22], combined with its unexplored function in EC, we hypothesized that PTBP1 might regulate ferroptosis in EC cells by post-transcriptionally modulating SLC7A11 expression.

This study is the first to identify PTBP1 as a key regulator of ferroptosis in EC. We demonstrate that PTBP1 is upregulated in EC and promotes tumor progression. Mechanistically, PTBP1 binds directly to SLC7A11 mRNA and stabilizes it, thereby enhancing SLC7A11 protein expression to inhibit ferroptosis. Our findings establish the PTBP1-SLC7A11 axis as a critical regulatory pathway in EC and reveal a promising therapeutic target.

## Materials and methods

### Cell culture, shRNA/plasmid transfection, and lentivirus transduction

Ishikawa EC cells (#CL-0823, Procell, Wuhan, China) and KLE EC cells (#STCC10610P, Servicebio, Wuhan, China) were cultured at 37 °C in Dulbecco’s Modified Eagle Medium (DMEM​​, Procell) supplemented with 10% ​​fetal bovine serum (FBS)​​ and 1% streptomycin-penicillin, in a 5% CO_2_ atmosphere.

The following short hairpin RNA (shRNA)​​/plasmid constructs were obtained from Miaoling Biology (Wuhan, China): pLV2-U6-PTBP1(human)-shRNA1-Puro (#P34690), pLV2-U6-PTBP1(human)-shRNA2-Puro (#P34723), pLV2-U6-PTBP1(human)-shRNA3-Puro (#P34718), pLV3-CMV-PTBP1(human)-3×FLAG-Puro (#P48152), pLV2-CMV-EGFP-SLC7A11(human)-Puro (#P38047, denoted as SLC7A11-ov), pLV2-U6-shRNA-control (shNC), and a nontarget vector containing a scrambled sequence. PTBP1-depleted EC cells were generated by transfecting a mixture of shRNA1, shRNA2, and shRNA3 at a 1:1:1 ratio (referred to as shPTBP1) into KLE and Ishikawa cells using Lipofectamine 3000, following the manufacturer’s protocol (#L3000001, ThermoFisher, Waltham, MA, USA). SLC7A11 expression restoration in PTBP1-depleted EC cells was achieved by co-transfecting shPTBP1 with SLC7A11-ov into KLE and Ishikawa cells. The production of shPTBP1 lentivirus was performed by Obio (Shanghai, China) by transfecting shPTBP1 along with two packaging plasmids, pMD2.G and psPAX2, into 293T cells at 60–70% confluence. A stable PTBP1-depleted Ishikawa cell line was established by infecting Ishikawa cells with the shPTBP1 lentivirus at varying multiplicities of infection (MOI) and selecting positive cells using puromycin (2 µg/mL).

### Specimens of patients with EC

Formalin-fixed primary endometrial tumor specimens (specifically, the endometrioid carcinoma subtype, *n* = 12) were collected from patients with EC who underwent curative resection at Jiaozuo City People’s Hospital, after obtaining informed consent. Paired non-cancerous endometrial tissues (*n* = 12) from the same patients served as controls. All human-related assays adhered to protocols approved by Jiaozuo City People’s Hospital Ethics Committee (No. 2024-017-K17). Written informed consent was obtained from all participants.​.

### In vivo assay

All animal procedures conformed to National Institutional Guidelines and were approved by The Jiaozuo City People’s Hospital Animal Care and Use Committee (No. 2023-002-H02). For xenograft generation in BALB/c nude mice (Beiyou Biology, Beijing, China), 200 µL of ​serum-free media containing Ishikawa or KLE EC cells transduced with either shPTBP1 or shNC lentivirus was subcutaneously injected into the right flank of 8-week-old mice. Each group consisted of five mice (*n* = 5 per group). The health status of the mice was monitored daily​​, and no mortality or ​​adverse health events​​ were observed during the experiment. After five weeks, the mice were sacrificed, and their xenografts were harvested for volume measurement (width^2^ × length × 1/2), weight assessment, and expression analysis (mRNA and protein). The Ethics Committee approved a maximum tumor volume of 1,200 mm^3^ for this study. Throughout the experiments, no animal exceeded this limit. Animals were euthanized immediately if tumors approached 1,200 mm³. Animal welfare considerations included minimization of suffering through the humane use of CO_2_. Additionally, research staff received specialized training in animal care and handling to ensure ethical and proficient execution of experimental procedures.

### Immunohistochemistry

Immunohistochemical (IHC) analysis was performed on 24 formalin-fixed samples of patients with EC and six mouse xenografts to assess PTBP1, Ki67 (cell cycle marker), and HSP27 (ferroptosis suppressor), following the method described by Fang et al. [26]. The following primary antibodies were used: rabbit anti-PTBP1 pAb (#12582-1-AP, 1:300, Proteintech, Wuhan, China), rabbit anti-Ki67 pAb (#GB111499, Servicebio, 1:900, Wuhan, China), and rabbit anti-HSP27 pAb (#18284-1-AP, 1:250, Proteintech). Secondary antibody incubation and color development were performed using the Immunohistochemistry Kit for rabbit primary antibody (#36312ES) as per the manufacturer’s instructions (Yeasen, Shanghai, China).

IHC detection of 4-hydroxynonenal (4-HNE), a marker of lipid peroxidation, was performed on formalin-fixed, paraffin-embedded tissue sections. After deparaffinization, rehydration, and antigen retrieval, sections were incubated overnight at 4 °C with a rabbit anti-4-HNE polyclonal antibody. Subsequent steps, including secondary antibody incubation and color development, were performed using the Immunohistochemistry Kit for rabbit primary antibody according to the manufacturer’s instructions.

### Extraction of RNA, synthesis of cDNA, and quantitative PCR (qPCR)

RNA extraction from treated KLE and Ishikawa EC cells and mouse xenograft tissues was performed following the manufacturer’s guidelines (Qiagen, Milan, Italy) using the RNAeasy Kit (#1YM868). cDNA was synthesized from RNA with concentrations ranging from 5.6 to 6.8 ng/µL using the PrimeScript RT Kit (Takara, Beijing, China) and oligo(dT)_15_ primers. The cDNA was diluted 20-fold and subjected to real-time qPCR using SYBR Premix ExTaq II (Takara) and primers listed in Supplementary Table 1. The 2^–ΔΔCt^ method was used to calculate gene expression, with normalization to β-actin as a reference control. Each experiment included three independent biological replicates.​.

### Western blot

Total protein from treated EC cells and mouse xenograft tissues was extracted using the BBproExtra^®^ Total Protein Kit (#BB-3101) according to the manufacturer’s instructions (Bestbio, Suzhou, China). Immunoblotting was conducted with 20 µg of protein per lane as described by Kim et al. [27]. Primary antibodies used were: rabbit anti-PTBP1 pAb (#12582-1-AP, 1:5000, Proteintech), mouse anti-ACSL4 mAb (#66617-1-Ig, 1:8000, Proteintech), rabbit anti-SLC7A11 pAb (#Q9UPY5, 1:1000, Zenbio), rabbit anti-NOX1 pAb (#17772-1-AP, 1:3000, Proteintech), mouse anti-GPX4 mAb (#GB124327, 1:800, Servicebio), mouse anti-β-actin mAb (#ab6276, 1:10000, Abcam, Cambridge, UK), and horseradish peroxidase (HRP)-labeled anti-mouse (#ab6789) and anti-rabbit (#ab97051) IgG secondary antibodies (1:8000, Abcam). Densitometric analysis was performed after band visualization using electrochemiluminescence (ECL) reagent mix (Servicebio). Western blot data are presented as representative images from at least one experiment, and quantitative analyses were performed when multiple biological replicates were available.

### Cell viability assay

Cellular viability was assessed using the Enhanced Cell Counting Kit 8 (CCK8, #E-CK-A362, Elabscience, Wuhan, China) according to the manufacturer’s protocol. KLE and Ishikawa EC cells were transfected with shPTBP1, shPTBP1 + SLC7A11-ov, shNC, or shPTBP1 + vector-NC vectors. CCK8 solution was added to each well, and absorbance was measured at 450 nm. Each treatment group was assayed in five technical replicates.

### Measurement of reactive oxygen species (ROS) content

For ROS detection, KLE and Ishikawa EC cells were loaded with DCFH-DA probes using the ROS Assay Kit (#S0033S, Beyotime, Shanghai, China), following the manufacturer’s protocol. Briefly, 2’,7’-dichlorodihydrofluorescein diacetate​ (DCFH-DA) probes were diluted in serum-free media to a final concentration of 10 µM/L and added to each well for 20 min at 37 °C, followed by washing three times with fresh serum-free media. Each experiment included three independent biological replicates.​.

### Determination of contents of GSH, malondialdehyde (MDA), and Fe^2+^

MDA content was measured using the MDA Assay Kit (#S0131S, Beyotime), GSH levels were determined using the GSH Assay Kit (#ml094982, Enzyme-linked Biotechnology, Shanghai, China), and Fe^2+^ content was measured using the Ferrous Iron Colorimetric Assay Kit (#E-BC-K881-M, Elabscience), all following the provided protocols. Supernatants were collected from treated cells, and their protein concentrations were measured for quantification of GSH, MDA, and Fe^2+^. Each treatment group was assayed in three technical replicates.

### Immunofluorescence (IF)

IF microscopy for the detection of NOX1 and GPX4 was performed following a previously described method [28]. Briefly, KLE and Ishikawa EC cells with different treatments were first fixed with 4% formaldehyde, permeabilized using 0.5% Triton X-100, and blocked with 3% bovine serum albumin (BSA, Beyotime) to prevent non-specific binding. The cells were then probed overnight at 4 °C with mouse anti-GPX4 mAb (#67763-1-Ig, 1:900, Proteintech) or rabbit anti-NOX1 pAb (#ab55831, 1:100, Abcam). Alexa Fluor 488-labeled goat anti-mouse (#ab150113, 1:800, Abcam) or anti-rabbit (#ab150077, 1:1000, Abcam) secondary antibodies were used for visualization, producing green fluorescence. Nuclei were stained with DAPI, which forms a complex with DNA to produce blue fluorescence. IF analysis was performed on three independent biological replicates. For each replicate, images from three random fields of view were captured, and fluorescence intensity was quantified for statistical analysis.

### RNA sequencing (RNA-seq) and data analysis

Ishikawa EC cells transfected with shPTBP1 or shNC were used. cDNA libraries for RNA-seq were prepared from 500 ng of cellular RNA using the TruePrep RNA Library Prep Kit (#TR503, Vazyme, Nanjing, China) according to the manufacturer’s instructions. Illumina HiseqTM 2500 (Illumina, San Diego, CA, USA) was used to generate 150 base-pair reads. The number of reads mapped to human protein-coding genes in each sample was determined using the htseq-count software [29]. Differentially expressed genes (DEGs) between the two groups were identified using DESeq2 software. KEGG pathway enrichment analysis was performed on the DEGs using the KEGG database and hypergeometric distribution test.

### Prediction of targets of PTBP1 and luciferase reporter

To predict potential target RNA transcripts of PTBP1, the StarBase platform (https://rnasysu.com/encori/) was utilized. For the luciferase assay, SLC7A11 5’UTR or promoter reporters were constructed by inserting the SLC7A11 5’UTR (wild-type [WT] or harboring a mutated PTBP1 binding sequence) or the promoter region of the SLC7A11 gene into the PmeI and XbaI sites of the pmirGLO reporter construct (YouBio, Changsha, China). HEK-293T cells (#CL-0005, Procell) were cultured under conditions provided by Procell and used for this assay. The generated reporter constructs (WT or MUT) were co-transfected into HEK-293T cells with either shPTBP1 or shNC using Lipofectamine 3000 in 24-well culture plates. After 48 h, luciferase activity was measured using the Dual-reporter assay kit (#RG016, Beyotime), and the results were normalized to *Renilla* activity to control for transfection efficiency. Each experiment included three independent biological replicates.​.

### RNA Immunoprecipitation (RIP) and RNA pull-down assays

As previously described [30], two assays were performed to confirm the direct interaction between PTBP1 and SLC7A11. For RIP, the PureBinding^®^ RNA Immunoprecipitation Kit (#P0101) was used according to the manufacturer’s instructions (Generaybiotech, Beijing, China). Briefly, 1 µg of rabbit anti-PTBP1 pAb (#12582-1-AP, Proteintech) or isotype IgG control (#30000-0-AP, Proteintech) was added to lysates from Ishikawa EC cells and incubated for 8 h at 4 °C, followed by the addition of magnetic beads for an additional 2 h. The RNA bound to anti-PTBP1 or IgG immunoprecipitates was purified and analyzed for SLC7A11 mRNA enrichment using quantitative PCR. For the pull-down assay, the Pure Magnetic RNA-Protein Pull-down Kit (#RY6003, Writegene Biotechnology Co., Ltd., Zhengzhou, China) was used. Biotin-labeled SLC7A11 5’UTR or control probes (Tsingke, Xi’an, China) were incubated with pre-treated streptavidin beads in 100 µL of binding buffer for 30 min. After washing three times, Ishikawa EC cell lysates were added to the probe-bead complex and incubated overnight at 4 °C. The RNA-protein complex was collected, and the bound proteins were purified for PTBP1 enrichment analysis by Western blot. The RIP and RNA pull-down assays were performed with three independent biological replicates and a single experiment, respectively.

### Actinomycin D treatment for RNA stability

EC cells transfected with shPTBP1 and shNC controls were treated with 5 µg/mL actinomycin D (#S8964, Selleck, Shanghai, China). Cells were harvested at 0, 1, 2, 3, and 4 h post-treatment for quantitative PCR analysis of SLC7A11, ACSL4, and GPX4 mRNA. The statistical significance of mRNA decay over time between the shPTBP1 and shNC groups was determined using two-way analysis of variance (ANOVA).​ Each treatment group was assayed in three technical replicates.

### Statistical analysis

Statistical significance (*p < 0.05, **p < 0.01, and ***p < 0.001) was determined using Student’s *t*-test (two-tailed) or one-way/two-way ANOVA with Tukey’s test for multiple comparisons. Data are presented as means ± standard deviation.

## Results

### PTBP1 exerts oncogenic functions in endometrial cancer in vitro and in vivo​​

**Fig. 1:**
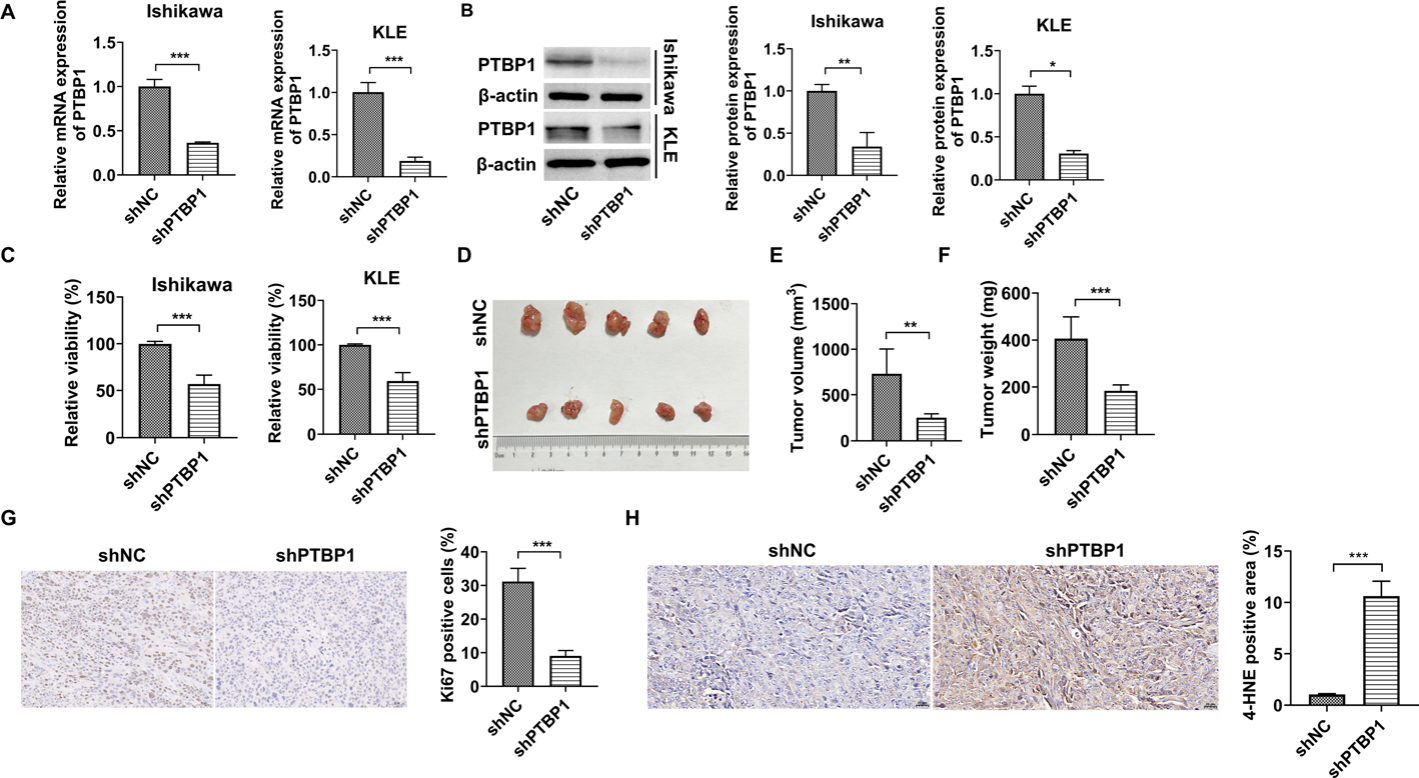
In vitro functional analysis of PTBP1 in endometrial cancer cells and in vivo validation using an Ishikawa xenograft model. **A** and **B** Transfection with shRNA knockdown vectors targeting PTBP1 significantly reduced both mRNA and protein levels in Ishikawa and KLE cell lines. **C** PTBP1 knockdown notably decreased cell viability in both Ishikawa and KLE cells. **D**–**H** Cell-derived xenograft (CDX) experiments in mice implanted with Ishikawa cells demonstrated that PTBP1 knockdown significantly reduced tumor volume and weight. Subsequent IHC staining of the xenograft tissues revealed a substantial decrease in Ki-67 protein levels and increase in 4-HNE level upon PTBP1 knockdown. *p < 0.05, **p < 0.01, ***p < 0.001

Previous studies have highlighted the tumor-promoting role of PTBP1 in various gynecological cancers, including breast and cervical cancers, as well as in other malignancies such as liver, colon, gastric cancers, and lung adenocarcinoma [21, 22]. However, the involvement of PTBP1 in EC remains largely unexplored. To address this gap, the significance of PTBP1 in EC was investigated through both in vitro and in vivo functional assays. Transfection of shRNA vectors targeting PTBP1 effectively reduced PTBP1 expression in Ishikawa and KLE cell lines (Fig. 1A and B) and substantially inhibited cell viability in these two EC cell lines (Fig. 1C). Cell-derived xenograft assays demonstrated that PTBP1 knockdown significantly suppressed tumor growth in vivo. ​​Specifically, results from Ishikawa cell xenografts (Fig. 1D and F) showed a significant reduction in tumor volume and weight, while parallel experiments using KLE cells (Supplementary Fig. 1A-1C) confirmed consistent antitumor effects. IHC analysis revealed that PTBP1 knockdown markedly reduced Ki67 protein levels in the corresponding xenograft tissues (Ishikawa: Fig. 1G; KLE: Supplementary Fig. 1D). These results collectively indicate that PTBP1 knockdown significantly suppresses the growth of endometrial cancer cells both in vitro and in vivo, suggesting that this gene plays a crucial role in the progression of endometrial cancer.

### PTBP1 protein is significantly upregulated in endometrial cancer

**Fig. 2:**
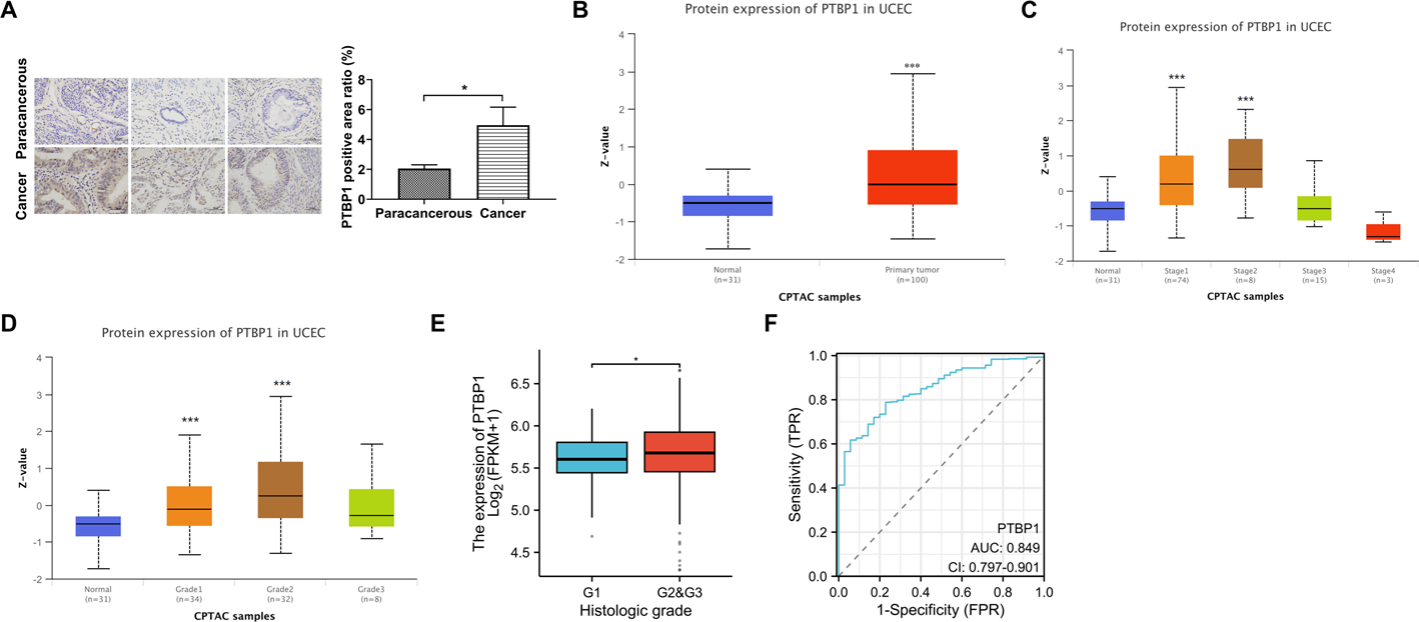
Clinical correlation between PTBP1 expression levels and endometrial cancer. **A** IHC analysis comparing PTBP1 protein levels in 12 paired endometrial carcinoma and adjacent normal tissue samples. **B** Comparative analysis of PTBP1 protein levels in endometrial carcinoma and normal endometrial tissues using CPTAC data from the UALCAN database. **C** Protein expression of PTBP1 across different tumor stages (Stage 1–4) in CPTAC samples. **D** Protein expression of PTBP1 across different tumor grades (Grade 1–3) in CPTAC samples. **E** The mRNA expression of PTBP1 related to tumor grades (Grade 1–3) in the TCGA database. **F** ROC curve evaluating the diagnostic potential of PTBP1 for endometrial cancer. *p < 0.05, ***p < 0.001

To further explore the clinical relevance of PTBP1 in EC, tumor and adjacent normal tissue samples were collected from 12 clinical donors. IHC analysis was performed to assess PTBP1 protein expression in these clinical samples. IHC results demonstrated that PTBP1 protein levels were significantly elevated in EC tissues compared to adjacent normal tissues in all 12 paired samples (Fig. 2A). Additionally, analysis of PTBP1 protein expression in EC tissues using CPTAC data from the UALCAN database confirmed a significant increase in PTBP1 levels in EC compared to normal tissues (Fig. 2B). We further investigated the association between PTBP1 expression and key clinicopathological parameters. Analysis of CPTAC data revealed that PTBP1 protein levels were significantly elevated in advanced tumor stages (Stage 1–2) compared to normal tissues (Fig. 2C). Moreover, a strong positive correlation was observed between PTBP1 expression and tumor grade, with significantly higher PTBP1 levels in Grade 1 and Grade 2 tumors compared to normal tissues in the CPTAC dataset (Fig. 2D). This grade-associated upregulation of PTBP1 was consistently validated in an independent cohort from TCGA database (Fig. 2E). To evaluate the diagnostic potential of PTBP1, we performed a ROC curve analysis. The result showed a high AUC of 0.849, indicating that PTBP1 possesses significant diagnostic value for distinguishing EC from normal endometrial tissues (Fig. 2F). These results collectively indicate that PTBP1 expression is markedly upregulated in EC, is associated with advanced disease progression, and exhibits strong diagnostic potential.

### PTBP1 disruption promotes ferroptosis in EC cells

**Fig. 3:**
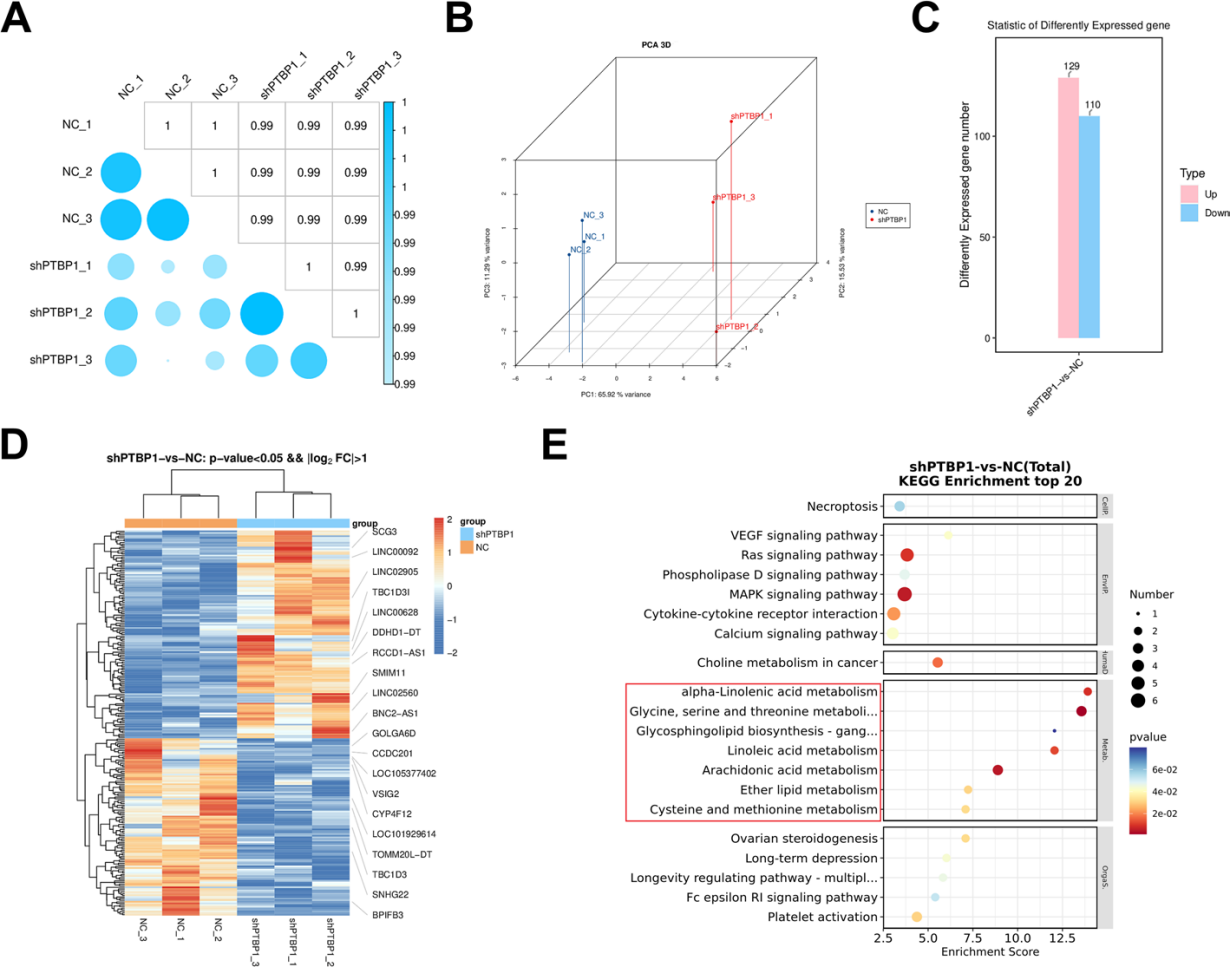
Identification of PTBP1-associated genes by RNA-seq. **A** and **B** Correlation coefficients test (**A**) and Principal Component Analysis (PCA) (**B**) of RNA-seq data for the two sample groups. **C** The upregulated and downregulated genes in Ishikawa EC cells after PTBP1 knockdown compared to the shNC control. **D** Heatmap showing the top 10 most significantly upregulated and downregulated genes in Ishikawa EC cells after PTBP1 knockdown. **E** KEGG pathway enrichment analysis of differentially expressed genes (DEGs) associated with PTBP1

To explore the molecular mechanisms underlying PTBP1’s role in EC progression, RNA sequencing was conducted to assess the global impact of PTBP1 knockdown on the transcriptome of EC cells (Fig. 3A and B). The sequencing data revealed that PTBP1 knockdown significantly upregulated the mRNA expression of 129 genes and downregulated the mRNA expression of 110 genes in EC cells (Fig. 3C and D). Functional enrichment analysis of these DEGs indicated significant involvement in biological processes related to lipid metabolism (Fig. 3E). Given the complex bidirectional relationship between lipid metabolism and ferroptosis in tumor cells [10, 31, 32], our findings suggest that PTBP1 may promote EC progression through regulation of ferroptosis.

### PTBP1 knockdown facilitates ferroptosis in endometrial cancer cells

**Fig. 4:**
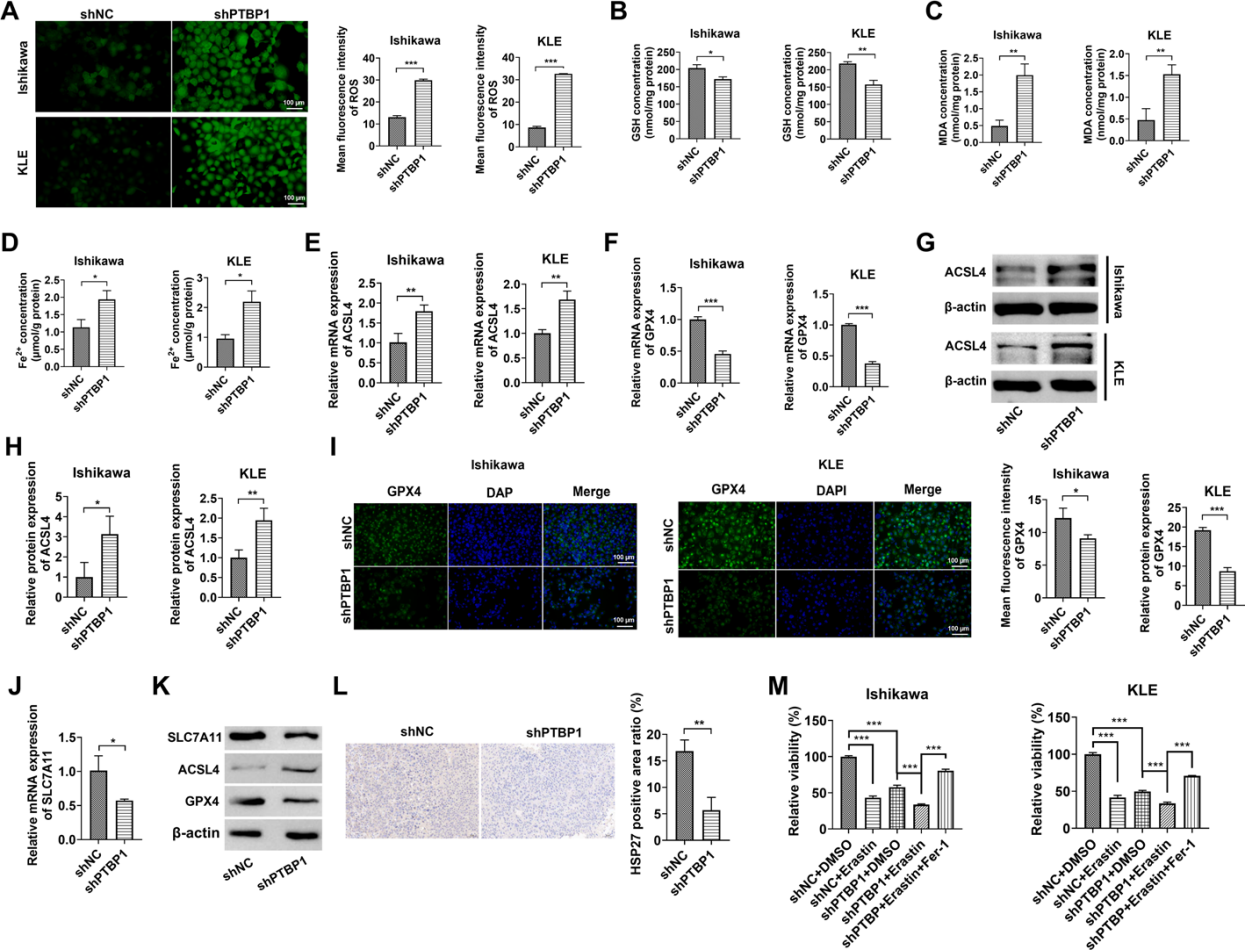
PTBP1 knockdown facilitates ferroptosis in endometrial cancer cells.**A** Measurement of ROS production using DCF-DA probes. **B** GSH, **C** MDA, and **D** Fe^2+^ levels in Ishikawa and KLE EC cells transfected with PTBP1-shRNA (shPTBP1) or nontargeting control (shNC) vectors, measured by relevant assay kits. **E–I** Ishikawa and KLE EC cells transfected with shPTBP1 or shNC for 48 h were analyzed for mRNA expression of ACSL4 (**E**) and GPX4 (**F**), by RT-qPCR, as well as protein expression of ACSL4 (**G** and **H**) by Western blot, and GPX4 (**I**) by immunofluorescence. **J** and **K** The mRNA expression of SLC7A11 (**J**), as well as protein expression of SLC7A11, ACSL4, and GPX4 (**K**) in mice tumor tissues were determined by RT-qPCR and western blot assays. **L** The protein level of HSP27 in mice tumor tissues was tested by IHC. **M** Cell viability of Ishikawa and KLE cells transduced with shNC or shPTBP1 and treated with DMSO, Erastin (10 µM), or Erastin plus Ferrostatin-1 (Fer-1, 1 µM) for 24 h, measured by CCK-8 assay. *p < 0.05, **p < 0.01, ***p < 0.001

To explore this further, the effects of PTBP1 knockdown on various ferroptosis biomarkers were examined in EC cell lines. The results showed that PTBP1 knockdown significantly increased ROS levels (Fig. 4A), reduced intracellular GSH concentrations (Fig. 4B), and elevated levels of both MDA and ferrous ions in Ishikawa and KLE cells (Fig. 4C and D). Additionally, qPCR, Western blot, and IF analyses revealed that PTBP1 knockdown significantly upregulated the expression of ACSL4, while downregulating the expression of GPX4 and HSP27 in Ishikawa and KLE cells (Fig. 4E and I). The expression levels of SLC7A11, ACSL4, GPX4, and HSP27 were also mediated by PTBP1 knockdown in nude mice tissue formed by Ishikawa (Fig. 4J and L) and KLE cells (Supplementary Fig. 1E and 1 F). To provide more direct evidence for the execution of ferroptosis in vivo, we assessed lipid peroxidation, which is a hallmark of ferroptosis, by IHC staining for 4-HNE in the Ishikawa or KLE xenograft tumors. Notably, PTBP1 knockdown led to a significant increase in 4-HNE accumulation (Fig. 1H and Supplementary Fig. 1G), indicating enhanced lipid peroxidation. To functionally validate this conclusion, we asked whether the molecular changes induced by PTBP1 knockdown would render cells more vulnerable to pharmacological induction of ferroptosis. We challenged the cells with the ferroptosis inducer Erastin. As shown in Fig. 4M, PTBP1 knockdown alone reduced cell viability, and PTBP1-deficient cells exhibited a significantly enhanced sensitivity to Erastin treatment compared to shNC controls. Crucially, this enhanced cell death was effectively rescued by co-treatment with the ferroptosis inhibitor Ferrostatin-1, providing direct pharmacological evidence that PTBP1 loss potentiates ferroptotic cell death in EC cells. These results suggest that PTBP1 knockdown may promote ferroptosis in EC cells and tumor tissues.

To confirm that the observed ferroptotic effects were specifically due to the loss of PTBP1 rather than off-target effects, we performed a rescue experiment by re-expressing an shRNA-resistant PTBP1 cDNA in PTBP1-knockdown EC cells. As shown in Supplementary Fig. 2A, the re-introduction of PTBP1 successfully restored its own expression and, importantly, rescued the protein level of SLC7A11. Consequently, the ferroptosis phenotypes induced by PTBP1 knockdown, including decreased cell viability (Supplementary Fig. 2B), elevated ROS (Supplementary Fig. 2C), GSH depletion, and the accumulation of MDA and Fe^2+^ (Supplementary Fig. 2D-F), were all significantly reversed. These data provide compelling evidence that PTBP1 functions as a specific suppressor of ferroptosis in EC cells.​.

### PTBP1 mediates mRNA stability of SLC7A11, a well-known suppressor of ferroptosis

**Fig. 5:**
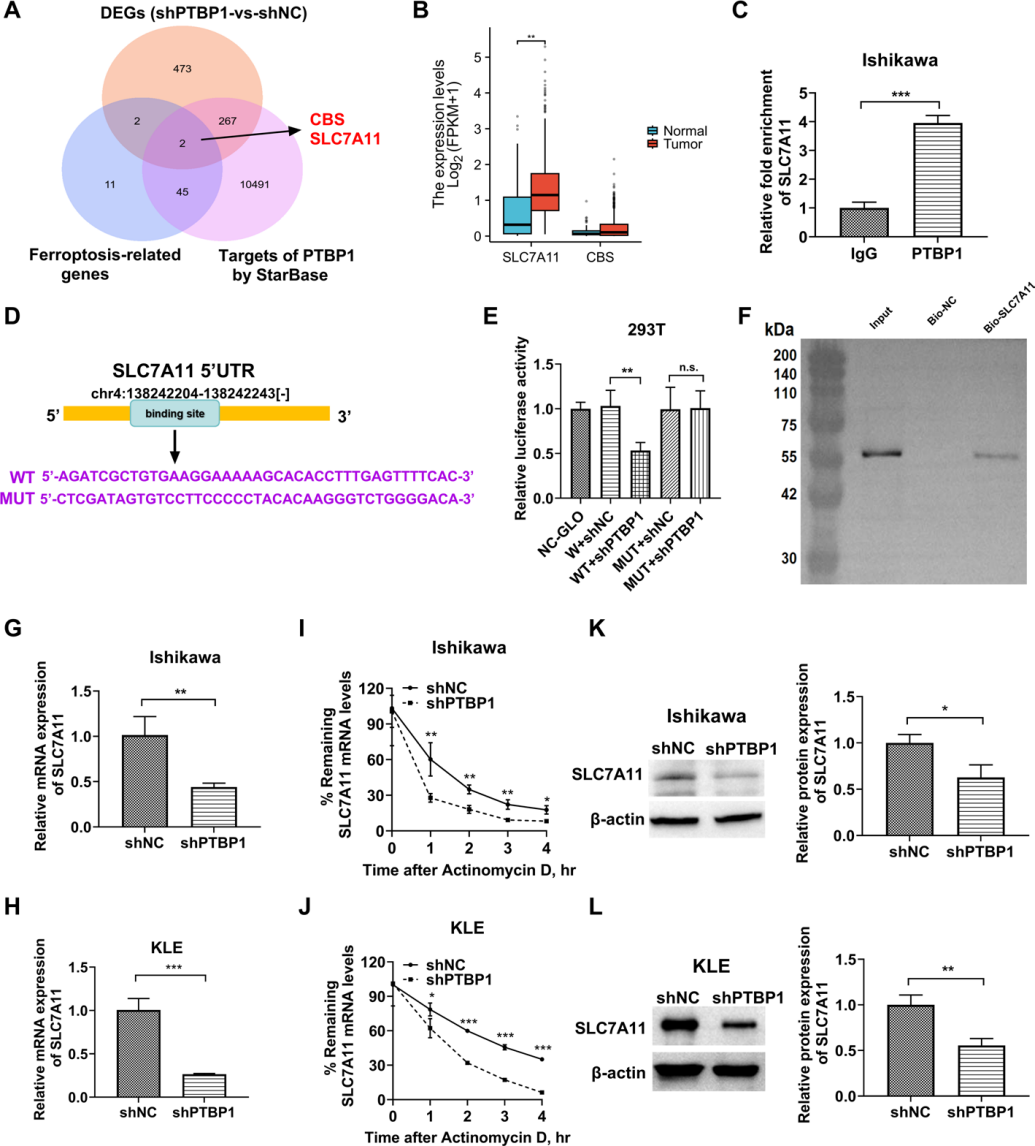
PTBP1 mediates SLC7A11 mRNA stability by binding to its 5’UTR. **A** Venn diagram displaying the overlap between differentially expressed genes (DEGs) (using criteria of *p*-adj < 0.05 and |log_2_FoldChange| >0.58) in PTBP1-KD Ishikawa EC cells versus shNC controls, ferroptosis-related genes, and predicted PTBP1 targets identified by the StarBase algorithm. **B** Transcriptomic analysis of TCGA revealed the mRNA expression levels of SLC7A11 and CBS in EC tissues compared to normal tissues. **C** Quantitative PCR analysis showing enrichment of SLC7A11 mRNA in anti-PTBP1 immunoprecipitates compared to anti-IgG controls. **D** Schematic representation of the SLC7A11 5’UTR, the predicted PTBP1 binding site (WT), and the mutated binding site (MUT). **E** Relative luciferase activity of WT or MUT reporters in shPTBP1- or shNC-transfected 293T cells. **F** DNA pull down-western blot showing PTBP1 enrichment in Bio-SLC7A11 5’UTR pull-downs compared to Bio-NC controls. **G** and **H** Quantification of SLC7A11 mRNA in shPTBP1- or shNC-transfected Ishikawa and KLE cells. **I** and **J** mRNA stability analysis using the actinomycin D exposure method, with RNA collected at 0, 1, 2, 3, and 4 h after exposure and quantified by PCR. **K** and **L** Measurement of SLC7A11 protein levels in PTBP1 KD and control Ishikawa and KLE cells. *p < 0.05, **p < 0.01, ***p < 0.001, n.s. non-significant

Previous studies have proposed the molecular mechanism by which PTBP1, as an RBP, promotes cancer progression by stabilizing the mRNAs of key oncogenes in cancer cells [22, 33]. Thus, PTBP1 may regulate ferroptosis in EC cells through a similar mechanism. A list of genes corresponding to mRNAs potentially interacting with PTBP1 was retrieved from the Starbase database, alongside a known ferroptosis-related gene list. Intersections between these two lists and the DEGs from our transcriptomic sequencing data following PTBP1 knockdown were analyzed. Notably, CBS and SLC7A11 were found at the intersection of these three gene sets (Fig. 5A). Clinical data indicate that SLC7A11 is aberrantly expressed in EC [34], and experimental evidence suggests that its depletion enhances ferroptosis in EC cells [35]. Transcriptomic analysis of data from The Cancer Genome Atlas (TCGA) revealed significantly elevated SLC7A11 expression in EC tissues compared to normal tissues, with no significant difference in CBS expression (Fig. 5B). Based on this, we investigated whether PTBP1 regulates SLC7A11. To examine this, the fold enrichment of SLC7A11 mRNA in anti-PTBP1 immunoprecipitations, compared to anti-IgG controls, revealed approximately a four-fold increase in SLC7A11 enrichment (Fig. 5C), supporting the interaction between PTBP1 and SLC7A11 mRNA. The StarBase algorithm predicted a single binding motif (5’-AGATCGCTGTGAAGGAAAAAGCACACCTTTGAGTTTTCAC-3’) within the 5’ UTR of SLC7A11 (Fig. 5D). To validate this, the SLC7A11 5’ UTR was cloned into a reporter plasmid (WT) for luciferase assays, which showed a significant reduction in luciferase activity following PTBP1 knockdown (Fig. 5E). In contrast, luciferase assays with a mutant reporter (MUT), in which all binding sites were mutated (Fig. 5E), revealed minimal influence of PTBP1 knockdown, confirming the specificity of the binding site for the PTBP1/SLC7A11 interaction. Furthermore, RNA pull-down experiments using biotin-labeled SLC7A11 5’ UTR probes further validated the direct interaction between PTBP1 and SLC7A11 in EC cells (Fig. 5F).

This study further tested the hypothesis that PTBP1 regulates the stability of SLC7A11 mRNA. mRNA expression analysis revealed a reduction in SLC7A11 levels following PTBP1 knockdown (Fig. 5G and H). To assess the stability of SLC7A11 mRNA in PTBP1-depleted EC cells, the actinomycin D method [36], which represses RNA synthesis, was used. The half-life of SLC7A11 mRNA was significantly shortened upon PTBP1 disruption (Fig. 5I and J), and SLC7A11 protein levels were also reduced in PTBP1-depleted EC cells (Fig. 5K and L). These data demonstrate that PTBP1 plays a key role in stabilizing SLC7A11 mRNA. Given that qPCR results indicated PTBP1 may regulate the mRNA expression of ACSL4 and GPX4, the impact of PTBP1 on the stability of these mRNAs was further assessed using the actinomycin D assay. The findings demonstrated that PTBP1 had no significant effect on the stability of ACSL4 and GPX4 mRNAs (Supplementary Fig. 3A and 3B). Previous studies have identified NRF2 and p53 as key regulators of SLC7A11 [37, 38], suggesting that PTBP1-mediated regulation of SLC7A11 mRNA stability could interact with these pathways. To explore this, the effects of PTBP1 overexpression on SLC7A11 mRNA levels were examined in wild-type, NRF2-overexpressing, or p53-overexpressing cells. The results showed that PTBP1 overexpression synergistically increased SLC7A11 mRNA levels in the presence of NRF2 overexpression, whereas p53 overexpression had no significant effect on SLC7A11 mRNA levels (Supplementary Fig. 3C). Given that NRF2 directly activates SLC7A11 transcription *via* its promoter [39], this study further assessed whether PTBP1 modulates NRF2’s effect on the SLC7A11 promoter using dual-luciferase reporter assays. The data indicated that NRF2 overexpression significantly enhanced luciferase activity driven by the SLC7A11 promoter, while PTBP1 overexpression had no substantial impact on this process (Supplementary Fig. 3D). These results further suggest that PTBP1 predominantly exerts its effects at the post-transcriptional stage, specifically through the regulation of mRNA stability, rather than at the transcriptional stage.

### PTBP1 disruption promotes EC cell ferroptosis through repression of SLC7A11

**Fig. 6:**
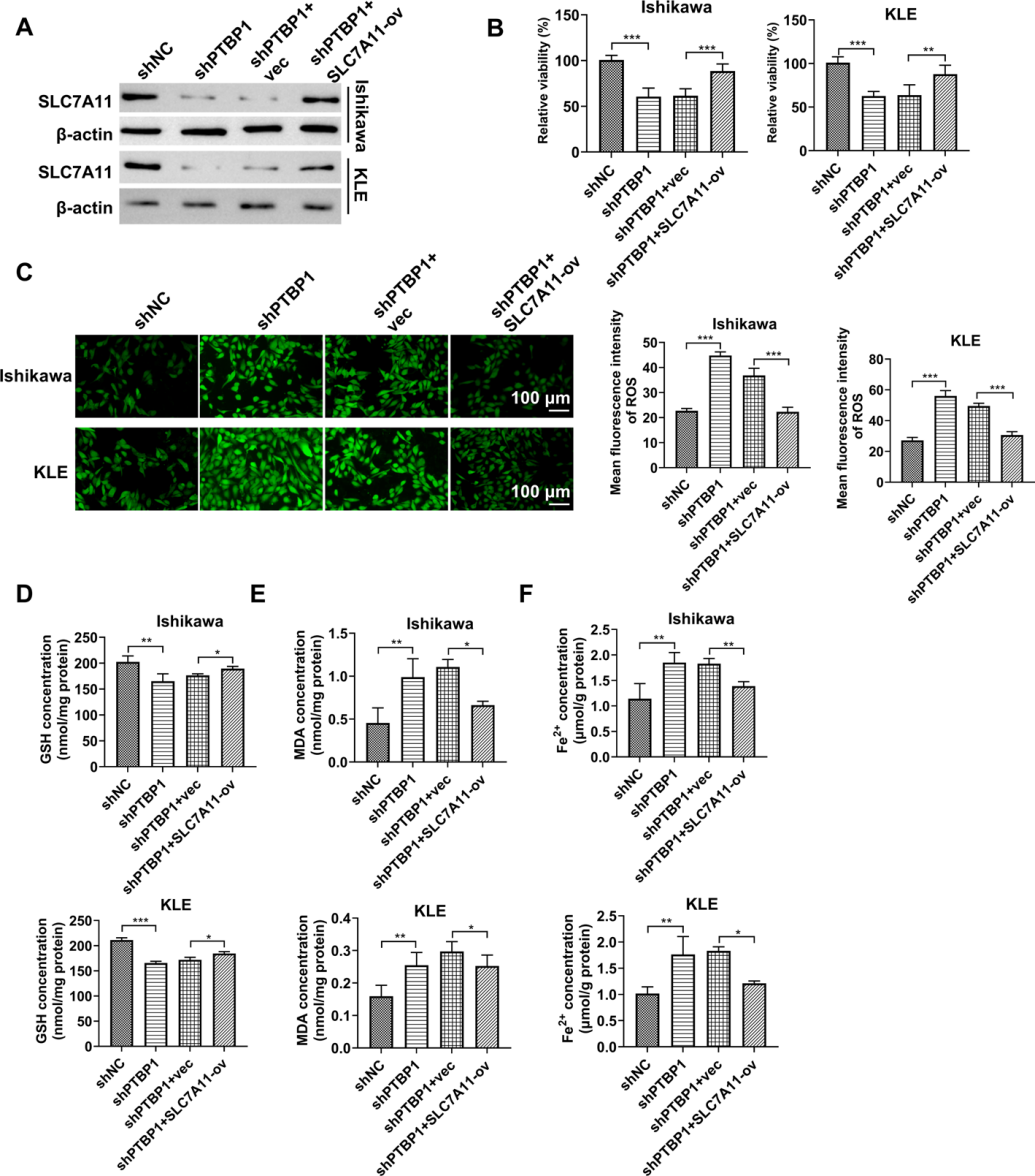
PTBP1 disruption induces ferroptosis through downregulation of SLC7A11. **A**–**F** Western blot analysis of SLC7A11 protein (**A**), CCK8 assay for cell viability (**B**), ROS production measured using DCFH-DA probes and fluorescence analysis (**C**), GSH content (**D**), MDA expression (**E**), and Fe2 + levels (**F**) in Ishikawa and KLE EC cells transfected with shPTBP1, shPTBP1 + SLC7A11-ov, shPTBP1 + vec, or nontargeting control shNC. *p < 0.05, **p < 0.01, ***p < 0.001

Our findings confirm that PTBP1 disruption promotes ferroptosis in EC cells. SLC7A11 depletion has been shown to enhance ferroptosis in EC cells [35], and the functional similarity between SLC7A11 and PTBP1 regulation of its mRNA stability prompted us to investigate whether PTBP1 modulates ferroptosis in EC cells through SLC7A11. To test this, SLC7A11 expression was restored in PTBP1-depleted Ishikawa and KLE EC cells. Co-transfection with an SLC7A11 ORF plasmid (SLC7A11-ov) significantly increased SLC7A11 protein levels in PTBP1-depleted cells (Fig. 6A). Cell viability, which was reduced in PTBP1 knockdown cells, was partially but significantly rescued by SLC7A11 restoration (Fig. 6B). Additionally, PTBP1 knockdown-induced ROS production was substantially diminished by restored SLC7A11 expression (Fig. 6C). PTBP1 knockdown also decreased GSH levels and increased MDA and Fe^2+^ concentrations in both EC cell lines, with these effects markedly reversed by SLC7A11 restoration (Fig. 6D-F). These results support the hypothesis that PTBP1 disruption induces ferroptosis in EC cells through the downregulation of SLC7A11.

### Knockdown of SLC7A11 alone is sufficient to induce ferroptosis in endometrial cancer cells

**Fig. 7:**
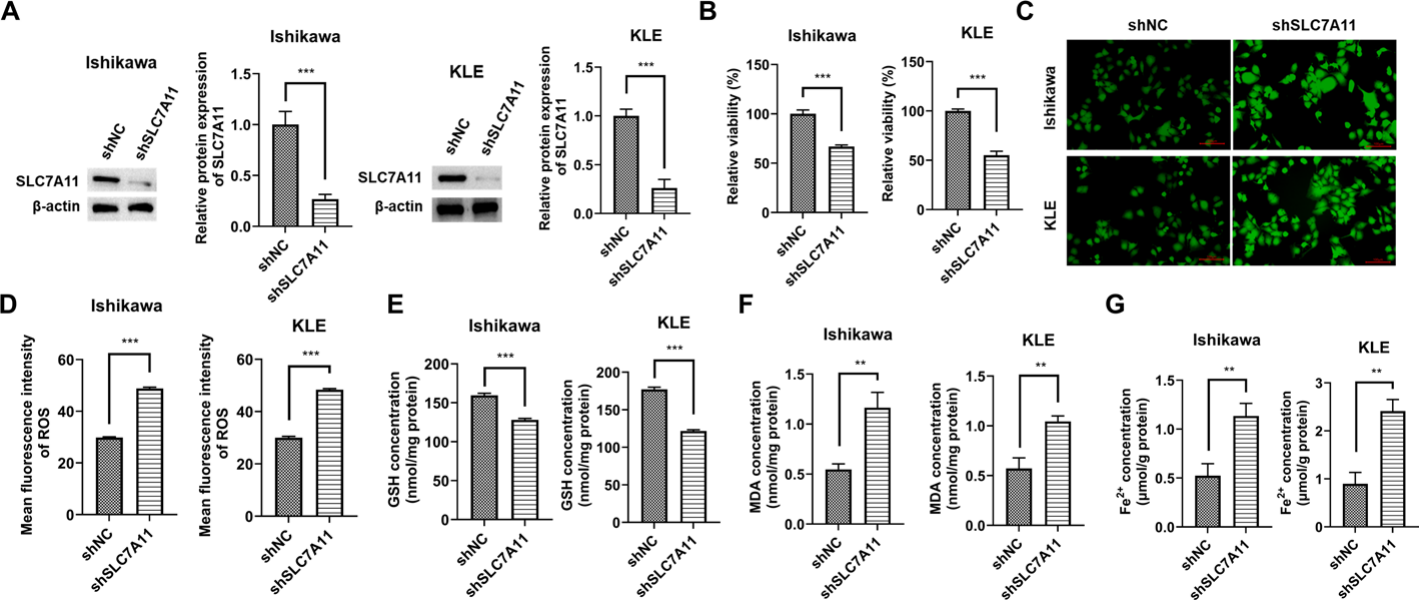
Knockdown of SLC7A11 alone is sufficient to induce ferroptosis in endometrial cancer cells. **A** Western blot analysis validating the knockdown efficiency of SLC7A11 in Ishikawa and KLE cells transfected with shSLC7A11 or shNC. **B** Cell viability measured by CCK-8 assay in Ishikawa and KLE cells after SLC7A11 knockdown . **C** and **D** Intracellular ROS levels detected by DCFH-DA fluorescence in Ishikawa and KLE cells after SLC7A11 knockdown. **E**–**G** Assessment of key ferroptosis biochemical markers in Ishikawa and KLE cells after SLC7A11 knockdown, including **E** glutathione (GSH) levels,​ **F**​ malondialdehyde (MDA) content, and **G** ferrous iron (Fe^2+^) concentration. **p < 0.01, ***p < 0.001

To definitively establish the necessity of SLC7A11 in suppressing ferroptosis in EC cells, we independently knocked down SLC7A11 without perturbing PTBP1. As shown in Fig. 7A, SLC7A11 was efficiently depleted in both Ishikawa and KLE cells. Strikingly, sole knockdown of SLC7A11 was sufficient to recapitulate the ferroptotic phenotype observed upon PTBP1 depletion. Specifically, SLC7A11 knockdown led to a significant reduction in cell viability (Fig. 7B), a marked increase in ROS accumulation (Fig. 7C and D), a profound depletion of GSH (Fig. 7E), and a significant elevation in both MDA (Fig. 7F) and intracellular Fe^2+^ levels (Fig. 7G). These results demonstrate that SLC7A11 is not merely a downstream target but is necessary for maintaining ferroptosis resistance in EC cells. Together with the rescue data in Fig. 6, these findings solidify the PTBP1-SLC7A11 axis as a critical regulatory pathway of ferroptosis in EC.

## Discussion

As a distinct form of cell death, ferroptosis has garnered significant research attention due to its involvement in various cancer contexts, where its induction could inhibit cancer progression by impeding aggressive cell behaviors and promoting cell death [32, 40]. To exploit ferroptosis as a therapeutic strategy, ongoing studies aim to identify molecules that either promote or inhibit ferroptosis in cancer cells [8, 40].

PTBP1 has been identified as an oncogene in multiple cancer types, contributing to malignant progression through several molecular mechanisms. For instance, PTBP1 regulates key gene expression *via* alternative splicing, influencing processes such as tumor proliferation, migration, invasion, and apoptosis [22]. It also drives cancer-specific Warburg effects by modulating the splicing of the glycolytic enzyme PKM (PKM1 → PKM2) [41]. Additionally, PTBP1 mRNA levels correlate with immune infiltration, mutation burden, treatment response, and prognosis in various cancers [42–44]. However, the molecular mechanisms by which PTBP1 influences EC remain largely unexplored. This study reports for the first time that PTBP1 expression is significantly elevated in EC. Both in vitro and in vivo functional assays confirmed the tumor-promoting role of PTBP1 in EC.

Our findings further demonstrate that PTBP1 depletion enhances ferroptosis in Ishikawa and KLE EC cells, suggesting an anti-cancer effect of PTBP1 depletion in EC. Notably, our results highlight the involvement of SLC7A11, a key ferroptosis suppressor, in the ability of PTBP1 to regulate ferroptosis in EC cells. These observations suggest that the PTBP1-SLC7A11 axis governs ferroptosis in EC cells and could offer a promising strategy for combating EC progression through ferroptosis-related approaches. Specifically, targeting PTBP1 could be explored as a potential strategy to sensitize EC cells to existing ferroptosis inducers (e.g., erastin, RSL3). Given the role of SLC7A11 in mediating ferroptosis resistance, its downregulation via PTBP1 inhibition might lower the threshold for ferroptosis induction. This suggests that PTBP1 inhibition, perhaps in combination with ferroptosis inducers, might warrant further investigation for EC treatment, especially in tumors exhibiting high PTBP1/SLC7A11 expression. Furthermore, assessing PTBP1 levels might help identify patient subgroups that could potentially benefit from such combination strategies, paving the way for future research into personalized therapies aimed at inducing ferroptosis in EC.

PTBP1 plays a pivotal role in mediating human carcinogenesis by regulating various aspects of cancer cell behavior, including growth, apoptosis, metastasis, and glycolysis [22]. In addition to these functions, accumulating evidence highlights PTBP1’s involvement in lipid metabolism. For instance, Yu et al. demonstrated that the stabilization of PTBP1 by circRNA hsa_circ_0007334 drives lipid metabolism reprogramming, contributing to the development of intrahepatic cholangiocarcinoma [45]. Similarly, in EC, PTBP1 induces lipid metabolism dysregulation, thereby promoting disease progression [46]. Given that lipid metabolism is critical for ferroptosis, with lipid peroxidation being a key trigger of this process [31], these findings suggest that PTBP1 could act as a potential regulator of ferroptosis. To explore this hypothesis, the role of PTBP1 in ferroptosis regulation was investigated in EC cells by depleting PTBP1 in Ishikawa and KLE EC cell lines. Our results indicate that PTBP1 depletion enhances ferroptosis in these cells, confirming PTBP1’s role as a suppressor of ferroptosis in EC. The ferroptosis regulators NOX1 and ACSL4 promote ferroptosis induction, while GPX4 and HSP27 act as potent suppressors of ferroptosis [31]. Our data on the levels of these factors support the notion that PTBP1 suppresses ferroptosis in EC cells. Notably, the observed ferroptosis enhancement upon PTBP1 depletion is partly due to the upregulation of NOX1 and ACSL4 and the downregulation of GPX4 and HSP27. These findings underline the complex role of PTBP1 in mediating ferroptosis. Interestingly, contrary to our conclusions, previous studies have shown that PTBP1 disruption in sorafenib-treated Huh-7 and HepG2 liver cancer cells significantly inhibits ferroptosis [25]. This apparent contradiction regarding the role of PTBP1 in ferroptosis between EC and liver cancer highlights the critical importance of ​​cell type-specific context in determining RBP function. The ultimate biological outcome of PTBP1 likely depends on the specific repertoire of mRNA targets it stabilizes or destabilizes in a given cellular environment. This functional plasticity is further evidenced by its roles in other cancers. In glioma cells, PTBP1 stabilizes PFKFB4 mRNA to promote glycolysis [47], whereas in gastric cancer cells, it accelerates the degradation of TXNIP mRNA to alleviate oxidative stress [48].​​.

Applying this principle to our findings, in EC cells, we identified SLC7A11 as a key target. PTBP1-mediated stabilization of SLC7A11 mRNA sustains cystine uptake and GSH synthesis, thereby exerting a strong anti-ferroptotic effect. In contrast, in liver cancer cells under sorafenib treatment, PTBP1 promotes the translation of NCOA4 mRNA, a key regulator of ferritinophagy, which sensitizes cells to ferroptosis [25]. This comparative analysis suggests a model wherein PTBP1 can function as either a suppressor or a promoter of ferroptosis, akin to its divergent roles in glycolysis and oxidative stress regulation, by engaging distinct mRNA targets, SLC7A11 for survival versus NCOA4 for death. The determinants of this target specificity may include cell-specific expression patterns of potential target mRNAs, the presence of competing RBPs, or post-translational modifications of PTBP1 itself. Our study, by delineating the PTBP1-SLC7A11 axis in EC, not only proposed a novel mechanism driving EC progression but also provides an example of the profound impact of cellular context on the function of RBP. Our data also show that PTBP1 is overexpressed in clinical EC specimens, suggesting its potential as a diagnostic marker for EC. However, this potential needs further validation through expanded analysis of PTBP1 expression in a larger cohort of EC samples.

As a key RBP, PTBP1 plays intricate roles in RNA splicing and metabolism, stabilizing multiple mRNAs, such as BECN1 and β-catenin [49, 50], thereby functioning as an RNA stabilizing regulator. SLC7A11 has been shown to drive resistance to ferroptosis, and its overexpression contributes to tumorigenesis by inhibiting ferroptosis [12]. SLC7A11 is highly expressed in various cancers, including EC [34], and its expression is associated with tumor microsatellite instability [51] and central carbon metabolism [52]. In the present study, PTBP1 stabilized SLC7A11 mRNA in EC cells by binding to its 5’UTR. A previous study by Cho and colleagues demonstrated that PTBP1 contributes to AXL mRNA degradation by targeting its 5’UTR in lung cancer cells [53]. These findings highlight PTBP1’s multifaceted roles in regulating mRNA stability. A critical question arising from our findings is the basis for the specific recognition of SLC7A11 mRNA by PTBP1, particularly given that the mRNA stability of other ferroptosis regulators like ACSL4 and GPX4 remained unaffected. While PTBP1 is known to bind pyrimidine-rich motifs, our luciferase and RNA pull-down assays point to the existence of a unique cis-element within the 5’UTR of SLC7A11 that is critical for this interaction. We speculate that the specific sequence context and secondary structural features of this binding platform in the SLC7A11 5’UTR confer a high affinity for PTBP1, a characteristic that appears to be absent or distinct in the UTRs of ACSL4 and GPX4. This proposed model provides a plausible mechanistic explanation for the selective regulation of SLC7A11. Future structural studies or transcriptome-wide approaches such as cross-linking immunoprecipitation sequencing (CLIP-seq) will be essential to precisely define this binding interface and comprehensively elucidate the rules governing PTBP1’s target selectivity in EC cells.​.

The depletion of SLC7A11 has been shown to promote ferroptosis in EC cells [35]. Our discovery that PTBP1 disruption induces ferroptosis through SLC7A11 depletion provides a molecular explanation for PTBP1’s role in repressing ferroptosis. In further support of this, our in vivo results demonstrate that PTBP1 depletion suppresses tumorigenesis in Ishikawa EC cells by inducing ferroptosis. Future studies are needed to confirm that the tumor-suppressive effect of PTBP1 depletion in EC is, at least partially, dependent on SLC7A11 downregulation.

It is important to acknowledge the limitations of our current study. While we have established a clear functional link between PTBP1, SLC7A11, and ferroptosis regulation in EC, our work primarily focuses on this specific axis. As an RNA-binding protein with a broad range of targets, it is plausible that PTBP1 may influence ferroptosis through additional mechanisms independent of SLC7A11. For instance, it could potentially regulate the expression or splicing of other key ferroptosis-related genes that were not explored here. Furthermore, while our RNA-seq data provided valuable insights, the study does not fully exclude the contribution of other PTBP1-regulated pathways to the observed phenotypes. Future investigations employing techniques such as transcriptome-wide profiling (CLIP-seq) of PTBP1-binding targets in EC cells will be crucial to uncover the full spectrum of its regulatory network and to determine the relative contribution of the SLC7A11-dependent and -independent pathways in mediating PTBP1’s anti-ferroptotic function.​.

In conclusion, PTBP1 was identified as a potent suppressor of ferroptosis in EC cells. Moreover, this study linked two well-established tumor drivers, PTBP1 and SLC7A11, through the post-transcriptional regulation of SLC7A11 by PTBP1. PTBP1 may serve as a promising therapeutic target and diagnostic marker for EC, and targeting PTBP1 expression could provide a novel approach to combating EC progression through ferroptosis-related strategies.

## Electronic Supplementary Material

Below is the link to the electronic supplementary material.

Supplementary Material 1. Supplementary Figure 1. PTBP1 knockdown inhibits tumor growth and modulates ferroptosis-related gene expression in KLE cell-derived xenograft models. (A-C) CDX (cell-derived xenograft) experiments demonstrated that PTBP1 knockdown significantly reduced tumor volume and weight in nude mice implanted with KLE. (D) IHC staining revealed that PTBP1 knockdown substantially decreased Ki-67 protein levels in tumor tissues from KLE in nude mice. (E and F) The mRNA expression of SLC7A11 (E), as well as protein expression of SLC7A11, ACSL4, and GPX4 (F) in mice tumor tissues were determined by RT-qPCR and western blot assays. (G) Representative IHC images and quantitative analysis of 4-HNE staining, a marker of lipid peroxidation, in tumor tissues from nude mice implanted with KLE cells transduced with shNC or shPTBP1. **p < 0.01, ***p < 0.001.

Supplementary Material 2. Supplementary Figure 2. Restoration of PTBP1 expression rescues ferroptosis phenotypes induced by PTBP1 knockdown. (A) Western blot analysis of PTBP1 and SLC7A11 protein levels in Ishikawa and KLE cells transfected with shNC+Vec, shPTBP1+Vec, or shPTBP1+PTBP1-Rescue. (B) Cell viability measured by CCK-8 assay in Ishikawa and KLE cells under the indicated conditions. (C) Intracellular ROS levels detected by fluorescence microscopy. (D-F) Assessment of key ferroptosis biochemical markers, including (D) GSH, (E) MDA, and (F) Fe2+ content. **p < 0.01, ***p < 0.001.

Supplementary Material 3. Supplementary Figure 3. Relationship between PTBP1 gene expression levels and other ferroptosis-related factors. (A and B) Actinomycin D assay to analyze the effect of PTBP1 knockdown on the stability of ACSL4 and GPX4 mRNAs. (C) RT-qPCR analysis to assess the effects of overexpression of PTBP1, NRF2, and P53 genes on SLC7A11 mRNA levels. (D) Dual-luciferase reporter gene assay examining the impact of PTBP1 or NRF2 overexpression on SLC7A11 promoter activity. ***p < 0.001.

Supplementary Material 4. Supplementary Table 1. Sequences of primers used for quantitative PCR.

Supplementary Material 5. Supplementary Table 2. DEGs between the shPTBP1 group and shNC control group.

## Data Availability

The RNA-seq datasets generated during the current study are available in the NCBI repository, under the accession number PRJNA1344470 (https://dataview.ncbi.nlm.nih.gov/object/PRJNA1344470).

## Abbreviations

PTBP1: Polypyrimidine tract-binding protein 1
EC: Endometrial carcinoma
RNA-seq: RNA sequencing
RBPs: RNA binding proteins
DEGs: Differentially expressed genes
shNC: shRNA-control
RT: Reverse transcription
shPTBP1: PTBP1-shRNA vector constructs
RBP: RNA-binding protein
PCA: principal component analysis
