# Supplementary figures and images for: PTBP1 functions as a suppressor of ferroptosis in endometrial carcinoma cells by stabilizing SLC7A11 mRNA
Source: Discov Oncol. 2025 Nov 28;16:2341. doi: 10.1007/s12672-025-04128-0 (PMC12753587; doi:10.1007/s12672-025-04128-0)

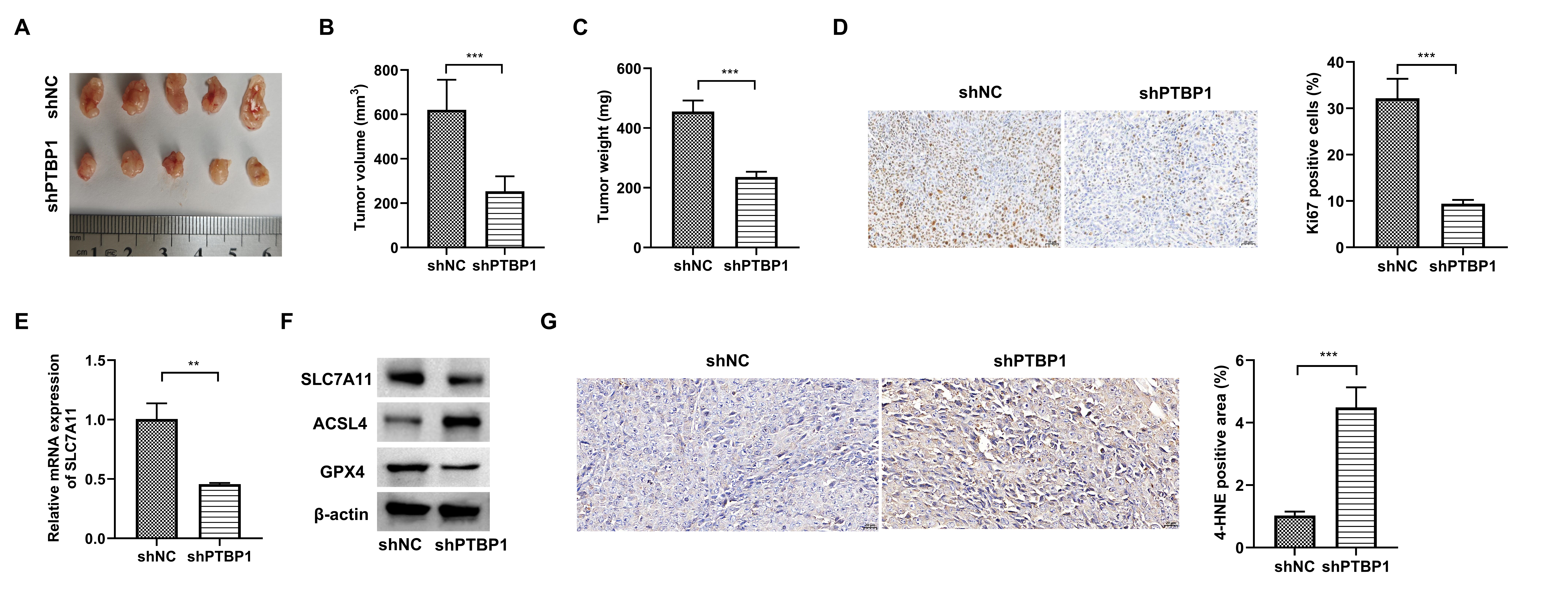

Supplement: Supplementary file 1 — Supplementary Material 1. Supplementary Figure 1. PTBP1 knockdown inhibits tumor growth and modulates ferroptosis-related gene expression in KLE cell-derived xenograft models. (A-C) CDX (cell-derived xenograft) experiments demonstrated that PTBP1 knockdown significantly reduced tumor volume and weight in nude mice implanted with KLE. (D) IHC staining revealed that PTBP1 knockdown substantially decreased Ki-67 protein levels in tumor tissues from KLE in nude mice. (E and F) The mRNA expression of SLC7A11 (E), as well as protein expression of SLC7A11, ACSL4, and GPX4 (F) in mice tumor tissues were determined by RT-qPCR and western blot assays. (G) Representative IHC images and quantitative analysis of 4-HNE staining, a marker of lipid peroxidation, in tumor tissues from nude mice implanted with KLE cells transduced with shNC or shPTBP1. **p < 0.01, ***p < 0.001. [file 12672_2025_4128_MOESM1_ESM.tif]

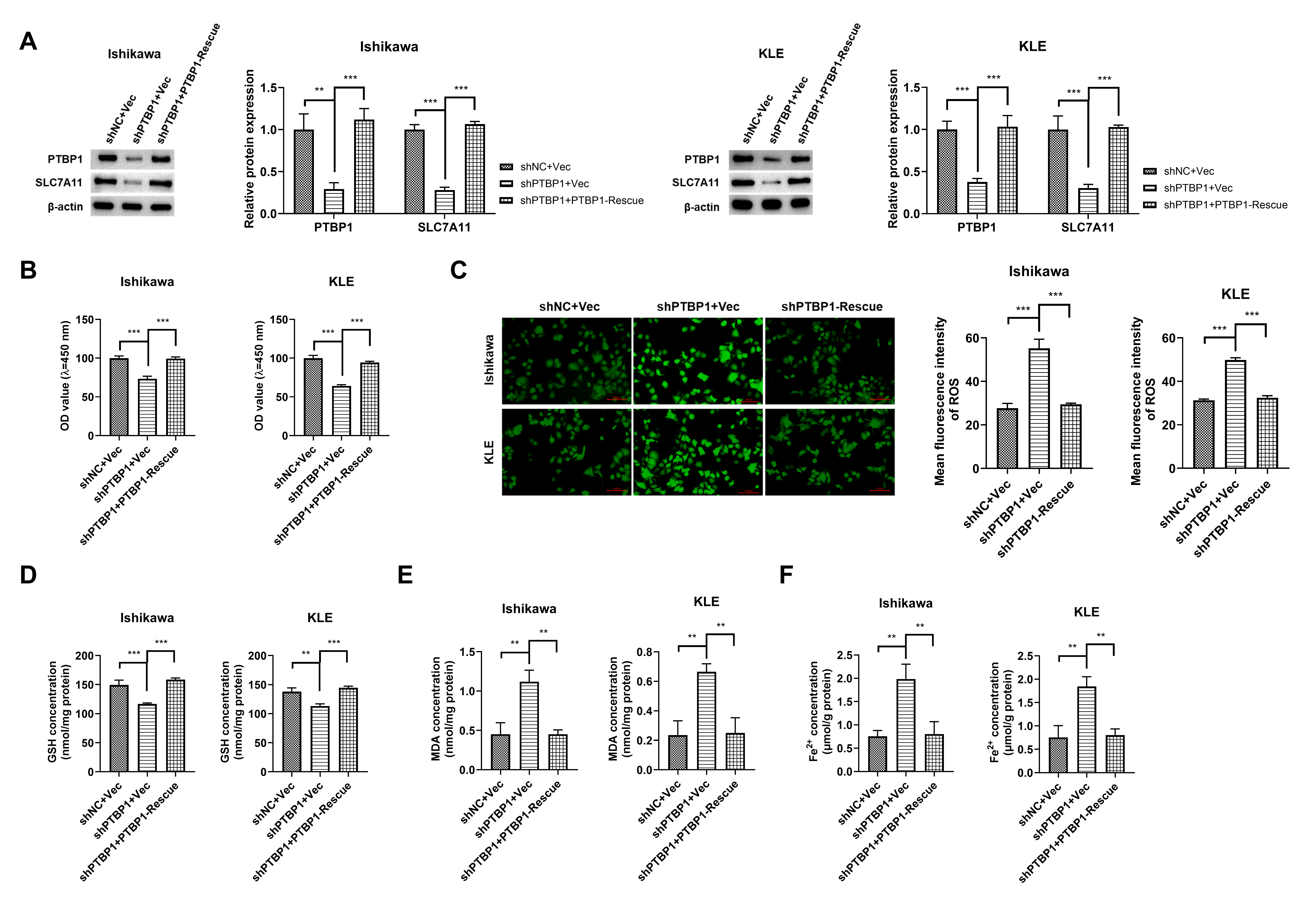

Supplement: Supplementary file 2 — Supplementary Material 2. Supplementary Figure 2. Restoration of PTBP1 expression rescues ferroptosis phenotypes induced by PTBP1 knockdown. (A) Western blot analysis of PTBP1 and SLC7A11 protein levels in Ishikawa and KLE cells transfected with shNC+Vec, shPTBP1+Vec, or shPTBP1+PTBP1-Rescue. (B) Cell viability measured by CCK-8 assay in Ishikawa and KLE cells under the indicated conditions. (C) Intracellular ROS levels detected by fluorescence microscopy. (D-F) Assessment of key ferroptosis biochemical markers, including (D) GSH, (E) MDA, and (F) Fe2+ content. **p < 0.01, ***p < 0.001. [file 12672_2025_4128_MOESM2_ESM.tif]

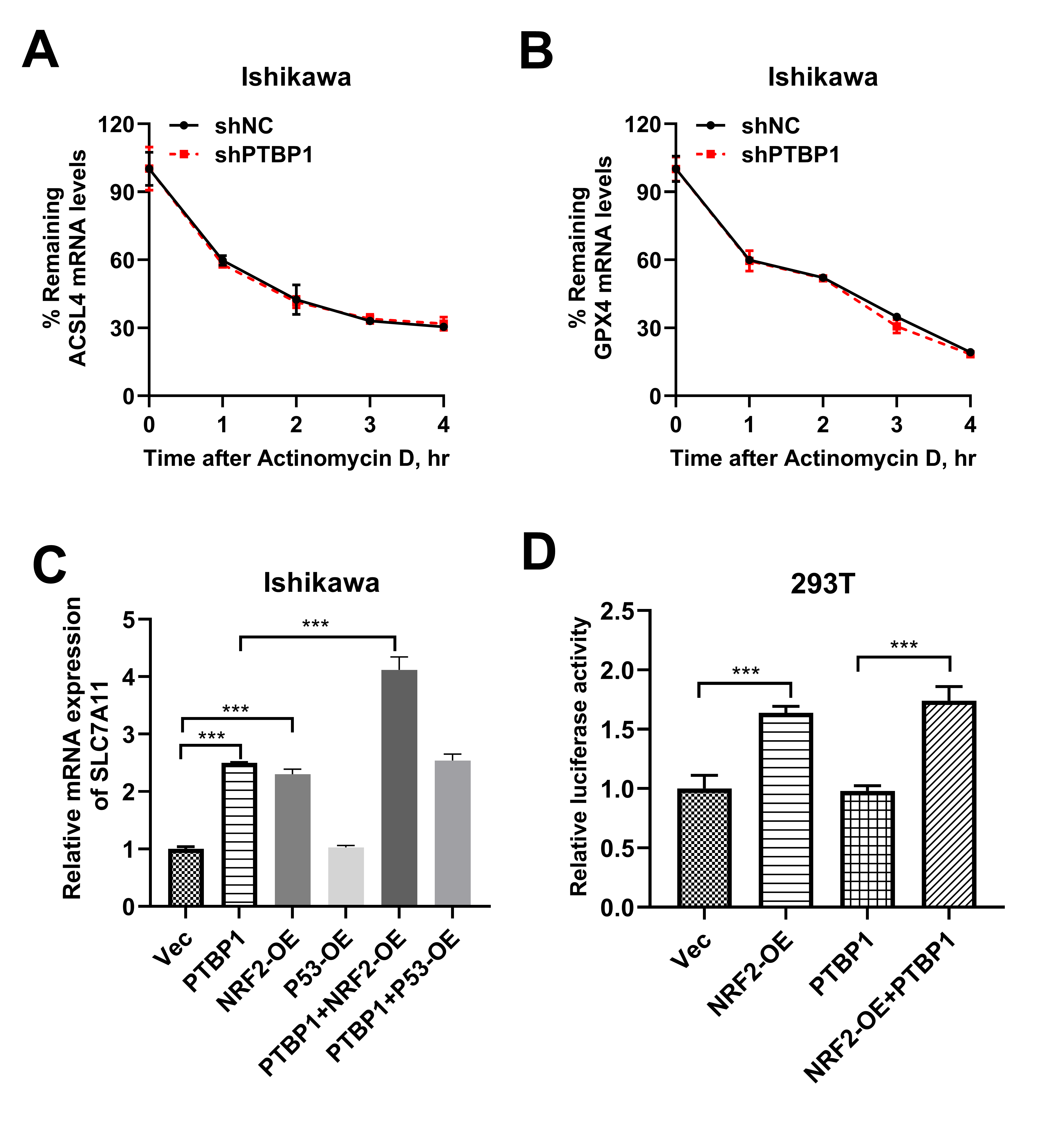

Supplement: Supplementary file 3 — Supplementary Material 3. Supplementary Figure 3. Relationship between PTBP1 gene expression levels and other ferroptosis-related factors. (A and B) Actinomycin D assay to analyze the effect of PTBP1 knockdown on the stability of ACSL4 and GPX4 mRNAs. (C) RT-qPCR analysis to assess the effects of overexpression of PTBP1, NRF2, and P53 genes on SLC7A11 mRNA levels. (D) Dual-luciferase reporter gene assay examining the impact of PTBP1 or NRF2 overexpression on SLC7A11 promoter activity. ***p < 0.001. [file 12672_2025_4128_MOESM3_ESM.tif]
