# Supplementary material for: PTBP1 functions as a suppressor of ferroptosis in endometrial carcinoma cells by stabilizing SLC7A11 mRNA
Source: Discov Oncol. 2025 Nov 28;16:2341. doi: 10.1007/s12672-025-04128-0 (PMC12753587; doi:10.1007/s12672-025-04128-0)
Supplement: Supplementary file 4 — Supplementary Material 4. Supplementary Table 1. Sequences of primers used for quantitative PCR. [file 12672_2025_4128_MOESM4_ESM.docx]

**Supplementary Table 1. Sequences of primers used for quantitative PCR**

| Name |  | Primers for PCR (5’-3’) |
| --- | --- | --- |
| PTBP1 | Forward | AGAACGCCCTAGTGCAGATG |
|  | Reverse | CACGTTCTGGTGCTTCGAGA |
| ACSL4 | Forward | CAGAATCATGTGGTGCTGGGAC |
|  | Reverse | ATTGTATAACCGCCTTCTTGCC |
| NOX1 | Forward | TCTGGTTGTTTGGTTAGGGCTG |
|  | Reverse | CGGCTGCAAAACCCAAGGA |
| GPX4 | Forward | CGGCGGAAGAAGCCCTG |
|  | Reverse | GTCGATGTCCTTGGCGGAAA |
| HSP27 | Forward | GCTACATCTCCCGGTGCTTC |
|  | Reverse | GATCTCGTTGGACTGCGTGG |
| SLC7A11 | Forward | GGTCAGAAAGCCTGTTGTGT |
|  | Reverse | GCACGCCCTTAGGAGAGATG |
| β-actin | Forward | GATTCCTATGTGGGCGACGA |
|  | Reverse | TCCCAGTTGGTGACGATGC |
